# A Small-Molecule Cocktails-Based Strategy in Culture of Mesenchymal Stem Cells

**DOI:** 10.3389/fbioe.2022.819148

**Published:** 2022-03-14

**Authors:** Yuan Feng, Jing Luo, Jintao Cheng, Aimin Xu, Dongbo Qiu, Sixiao He, Dayong Zheng, Changchang Jia, Qi Zhang, Nan Lin

**Affiliations:** ^1^ Department of Hepatobiliary Surgery, The Third Affiliated Hospital, Sun Yat-Sen University, Guangzhou, China; ^2^ Cell-gene Therapy Translational Medicine Research Center, The Third Affiliated Hospital, Sun Yat-Sen University, Guangzhou, China; ^3^ Department of Rehabilitation Medicine, The Third Affiliated Hospital, Sun Yat-Sen University, Guangzhou, China; ^4^ The First People’s Hospital of Kashi Prefecture, Kashi, China

**Keywords:** mesenchymal stem cells (MSC), small-molecule cocktails, cell viability, cell senescence, cell homogeneity

## Abstract

Mesenchymal stem cells (MSCs) have a variety of unique properties, such as stem cell multipotency and immune regulation, making them attractive for use in cell therapy. Before infusion therapy, MSCs are required to undergo tissue separation, purification, and expansion *in vitro* for a certain duration. During the process of *in vitro* expansion of MSCs, the influence of culture time and environment can lead to cell senescence, increased heterogeneity, and function attenuation, which limits their clinical applications. We used a cocktail of three small-molecule compounds, ACY (A-83–01, CHIR99021, and Y-27632), to increase the proliferation activity of MSCs *in vitro* and reduce cell senescence. ACY inhibited the increase in heterogeneity of MSCs and conserved their differentiation potential. Additionally, ACY maintained the phenotype of MSCs and upregulated the expression of immunomodulatory factors. These results suggest that ACY can effectively improve the quantity and quality of MSCs.

## Introduction

Mesenchymal stem cells (MSCs) have considerable application potential in the field of regenerative medicine due to their low immunogenicity, immunomodulatory, and self-renewal properties (Han et al., 2019). Because the efficiency of MSC recruitment to target sites is low (Zhang et al., 2018), a large number of MSCs are required for efficient therapy. MSCs are primarily isolated from tissues such as fat, umbilical cord, or bone marrow (Costa et al., 2021; Pittenger et al., 2019). However, the number of freshly isolated cells is insufficient for clinical applications. Therefore, large-scale *in vitro* expansion and culture methods are indispensable. Increases in cell culture generations and various influential factors in the culture environment, such as chemical compounds, radiation, and oxidative stress, can cause MSCs to become senescent and change their phenotype (Banimohamad-Shotorbani et al., 2020). As heterogeneity increased, the ability to proliferate and differentiate decreases markedly. Many studies have shown that it is challenging for MSCs to maintain stable therapeutic effects in clinical applications due to many non-directional changes in cellular characteristics and the upregulation of heterogeneity during the expansion process (Guo et al., 2006; Levy et al., 2020; Yin et al., 2019). There is a critical need for the development of MSC culture systems that reduce cell senescence and maintain long-term growth viability and cell homogeneity.

Some methods have been reported to improve the expansion and culture systems of MSCs, such as gene editing, to improve the targeting ability or the enhancement of paracrine effects from effective factors to ameliorate therapeutic effects (Levy et al., 2020; Nie et al., 2020; Ocansey et al., 2020). Additionally, biological materials and three-dimensional culture systems can also be used to maintain the primary phenotype of MSCs during the expansion process to conserve their growth activity (Lee et al., 2016; Drela et al., 2019; Gong et al., 2020). Induced pluripotent stem cells (iPSCs) are used for expansion and differentiation to obtain a high degree of homogeneity in MSCs, which avoids cell senescence issues (Ren et al., 2008; Sheyn et al., 2016; Fernandez-Rebollo et al., 2020). However, these methods still have their respective limitations, such as limited effect, cumbersome operation, and technical difficulty.

Studies have pointed out that the characteristics of MSCs can be altered by adding small-molecule compounds to the culture medium. For example, using TNF-α and IFN-γ to stimulate MSCs can promote homogeneity and improve the immune regulation function (Mohammadpour et al., 2015; Saldana et al., 2019). Addition of metformin can reduce the DNA damage of MSCs and slow cell aging (Jang et al., 2020; Kim et al., 2021; Zhang et al., 2021). We and others have reported that small molecules contribute to the fate regulation of proliferating and differentiating stem cells (Du et al., 2018; Hou et al., 2013; Katsuda et al., 2017). Moreover, we found that when inducing pluripotent stem cells to differentiate into hepatocytes, small-molecule compounds A-83–01 and CHIR99021 can effectively improve the multipotency and proliferation of hepatic stem cells, and Y-27632 can enhance the viability of resuscitated stem cells and reduce their apoptosis (Du C et al., 2018). These findings suggest that small-molecule compounds may help to maintain the proliferative activity and multipotency of other types of stem cells.

In this study, we developed a cocktail of three small-molecule compounds (A-83–01, CHIR99021, and Y-27632; ACY) to enhance the growth of MSCs and tested the function by evaluating the impact of this cocktail on cell viability and phenotypic properties of MSCs in cell culture. A-83–01 is an inhibitor of the TGF-β signaling pathway, CHIR99021 is an activator of the classic Wnt signaling pathway, and Y-27632 is an inhibitor of the ROCK signaling pathway. Our results revealed that ACY not only efficiently improved cell viability in MSCs but also exhibited a positive effect on the maintenance of the differentiation potential and phenotype of MSCs.

## Material and Methods

### Mesenchymal Stem Cells Isolation

Fat tissue-derived MSCs (FMSCs), bone marrow-derived MSCs (BMSCs), and umbilical cord-derived MSCs (UMSCs) used in this study were isolated from bone marrow aspirates, fat tissues, and umbilical cord from healthy donors following the Declaration of Helsinki protocols with informed consent. The information of donors is listed in Supplementary Table S1. The protocol was approved by the Clinical Research Ethics Committee of the Third Affiliated Hospital at Sun Yat-Sen University in Guangzhou, China.

For FMSCs isolation, fat tissues were isolated via liposuction and digested with 0.1% collagenase type I (Sigma-Aldrich) for 30 min at 37°C with gentle agitation. The collagenase was neutralized with low glucose DMEM containing 10% FBS. The digested aspirates were filtered through a 100-μm nylon cell strainer and cells were seeded at a density of 1 × 10^5^/cm^2^ into T75 cell culture flasks. Cells were cultured with the MSC medium containing low glucose DMEM (Gibco) supplemented with FBS (10%, Hyclone), fibroblast growth factor-basic (bFGF, 1 ng/ml, PeproTech), and penicillin/streptomycin (1%, Gibco). The first change of medium was accomplished within 12 h after isolation. Non-adherent cells were removed by a complete change of the medium, whereas the remaining adherent cells were cultured continuously. For BMSCs isolation, bone marrow aspirates were diluted with PBS and cells were isolated by density gradient centrifugation using Ficoll-Paque (Amersham Biosciences) and seeded at a density of 1 × 10^5^/cm^2^ into T75 cell culture flasks. The first change of medium was accomplished within 2 days after isolation. The same culture conditions and media were applied as described for FMSCs. For UMSCs isolation, the isolation protocol was performed as described in our previous research (Zhang et al., 2017). In brief, fresh human umbilical cords were obtained after birth and cut into 0.5 cm pieces and floated in MSC medium. The first change of medium was accomplished within 48 h after isolation. The same culture conditions and media were applied as described for FMSCs.

### Cell Culture and ACY Treatment

In an ACY (-) group, MSCs were cultured as previously described (Henrionnet et al., 2017; Samal et al., 2021). In brief, the culture medium contained low glucose DMEM (Gibco) supplemented with FBS (10%, Hyclone), fibroblast growth factor-basic (bFGF, 1 ng/ml, PeproTech), and penicillin/streptomycin (1%, Gibco). In ACY (+) group, the basal medium for culturing MSCs consisted of low glucose DMEM, FBS, bFGF, penicillin/streptomycin, and ACY cocktails consisted of A-83–01 (MedChemExpress), CHIR99021 (Selleck), and Y-27632 (Selleck). The medium was changed 1 day after seeding and every 2 days thereafter. There were 2 μM A-83–01, 3 μM CHIR99021, and 2 μM Y-27632 chosen as the optimal concentration in the ACY (+) group. When cells were approximately 90% confluent, they would be passaged with a split ratio of 1:3.

MSCs at passage 2 (P2) were seeded, without (ACY-) or treated (ACY+) with A-83–01, CHIR99021, and Y-27632, and were continuously cultured to passage 10 (P10; Supplementary Figure S1). From passage 5 (P5) to P10, the MSCs were used in subsequent analysis.

### Population Doubling Time Assay

MSCs (1 × 104 cells/cm2) were cultured in T25 cell culture flasks. The time was measured when cells were approximately 90% confluent.

### Cell Counting Kit-8 Assay

MSCs (1 × 103 cells) were cultured in a 96-well plate before the CCK-8 assay. Cells were continued to culture for 24, 72, 96, 120, and 168 h. CCK-8 reagent (DOJINDO) was added to cells according to the manufacturer’s instructions for cell activity measurement. The absorbance value at 450 nm on a microplate reader was recorded.

### Senescence-Associated *β*-galactosidase Staining Assay

SA-β-gal staining was performed as previously described. SA-β-gal staining was performed using an SA-β-gal Staining Kit (Beyotime) according to the manufacturer’s recommended protocol. Briefly, MSCs were fixed in paraformaldehyde (PFA, 4%) for 15 min and stained with SA-β-gal staining solution overnight. Cells with a bright blue color were considered positive.

### Flow Cytometry

Flow cytometric analyses were performed with Influx (BD Bioscience) or Gallios (Beckman Coulter) flow cytometers, and the data were analyzed with the FlowJo (Treestar). To examine the expression of MSCs markers, cells were incubated with anti-CD90, anti-CD105, anti-CD73, anti-CD44, anti-CD11B, anti-CD34, anti-CD19, anti-CD45, and anti-HLA-DR antibodies for 30 min at room temperature (BioLegend) according to the manufacturer’s recommended protocol. Antibody-positive populations were quantified and analyzed afterward.

### Karyotype and Telomerase Activity Analysis

MSCs were incubated in 20 μg/ml colcemid at 37°C for 30 min. Then, cells were dissociated with 0.125% trypsin, followed by incubation with Carnoy’s fixative solution. The G-banding karyotype of 30 randomly selected mitotic metaphase nuclei were analyzed.

For telomerase activity analysis, the Telomerase ELISA Assay Kit (MEIMIAN) was used according to the manufacturer’s protocol.

### Chemokines and Paracrine Factors Analysis

For chemokines and paracrine factors analysis, MSCs of P5 were treated with TNF-α (20 ng/ml) and IL-1β (20 ng/ml) for 24 h to stimulate the mRNA expression. After stimulation, the mRNAs were extracted, and qPCR was used for the following analysis. The primers are listed in Supplementary Table S2.

### Differentiation Analysis

For osteogenic, chondrogenic, and adipogenic differentiation of MSCs *in vitro*, we used the Osteogenic Differentiation Kit, Chondrogenic Differentiation Kit, and Adipogenic Differentiation Kit, purchased from Cyagen Biosciences and used as per manufacturer’s protocol. The staining intensity was quantitated and evaluated by ImageJ (National Institutes of Health).

### Analysis of Attached Cell Size

As previously described (Klinker et al., 2017), in brief, for morphological analysis, MSCs were seeded at a density of 10,000 cells per well (three total wells per experimental group) in 6-well plates and cultured for 24 h in ACY (-) or ACY (+) medium. Cell and nuclear morphology were assessed using fluorescein (FITC)-maleimide (Gibco) and Hoechst (Sigma-Aldrich), respectively. Briefly, cells were fixed in PFA for 15 min before incubated with FITC-maleimide (20 μM) diluted with Triton X-100 (0.1%, Thermo Fisher Scientific) for 30 min. Then, cells were washed with PBS, and incubated with Hoechst (1 mg/ml) for 5 min before imaging. Automated quantification of cellular and nuclear shape features was performed using CellProfiler (downloaded from https://cellprofiler.org/releases) (Carpenter et al., 2006) and ImageJ (National Institutes of Health) to obtain quantitative morphological data for each cell consisting of 30 of the most representative cellular shape features and 30 corresponding nuclear shape features.

### Mitochondrial Analysis

#### MitoTracker Staining

MSCs were incubated with MitoTracker staining solution (50 nM, Thermo Fisher Scientific) for 30 min. The MitoTracker staining solution was then removed, and the fresh cell culture solution was added to pre-incubate at 37°C. The subsequent observation was carried out with a fluorescence microscope. The volume and the mean (area/perimeter)/circularity index of the mitochondrial network was calculated using the Mito-Morphology macro in ImageJ.

#### JC-1 Staining

MSCs were washed with PBS, and JC-1 dyeing working solution (Beyotime) mixed with fresh medium was added. The cells were incubated at 37°C for 20 min in a cell incubator. After incubation at 37°C, the supernatant was aspirated and the cells were washed twice with JC-1 staining buffer (Beyotime). Afterward, 2 ml of cell culture medium was added, and the results were observed under a fluorescence microscope. Relative degrees of mitochondrial polarization were quantified by measuring the red-shifted JC-1 aggregates and the green-shifted monomers.

#### Reactive Oxygen Species Assay

DCFH-DA (Beyotime) was diluted with basal culture medium at a ratio of 1:1000 to a final concentration (10 μmol/L). Then, the cell culture medium was removed, and diluted DCFH-DA was added in an appropriate volume. The cells were incubated in a cell incubator at 37°C for 20 min. Then, the cells were washed three times with basal culture medium and the results were observed under a fluorescence microscope.

#### ATP Assay

MSCs (2.5 × 10^4^ cells) were plated in a 6-well plate and cultured overnight. The culture solution was aspirated, and 200 μL of lysis solution was added to every well of the 6-well plate to lyse the cells. Then, the lysis solution was centrifuged at 12,000 g at 4°C for 5 min, and the supernatant would be used for analysis. ATP detection working solution (Beyotime; 100 μL) was added to every detection hole and left it at room temperature for 3–5 min. Afterward, a sample or standard (20 μL) was added to every test hole, and Synergy H1 (BioTek) was used to detect the RLU value.

### Single Cell Transcriptome Analysis

#### Single Cell Capture and Sequencing

Single MSC was captured on a microfluidic chip, and nucleic acid-modified beads were used to molecularly label mRNAs from different cells, and finally a high-throughput single-cell transcriptome library compatible with Illumina Hiseq XTen sequencer was constructed. Fastp was used to trim primer sequence and low-quality bases of raw reads and collect the basic statistics. The cleaned reads after being trimmed were used to generate the transcript expression matrix.

#### Sequencing Data Quality Control

Fastp (v0.20.1) (Chen et al., 2018) was used to trim primer sequence and low-quality bases of raw reads and collect the basic statistics. The specific parameters are summarized below:

(1) A 4-bp sliding window was moved from front (5′) to tail. Once the mean quality of the bases in the window was below 10, the bases in the window, along with the subsequent bases, would be dropped, and the analysis within the read was finished. The leading N bases were also trimmed (--cut_front--cut_front_window_size 4 --cut_front_mean_quality 10).
(2) A 1-bp sliding window was moved from tail (3′) to front. The bases in the window were dropped if their mean quality was below 3, and the window kept moving until the last base. The trailing N bases were also trimmed, similar to the Trimmomatic TRAILING method (--cut_tail--cut_tail_window_size 1 --cut_tail_mean_quality 3).
(3) The auto adapter was detected for PE data (detect_adapter_for_pe).
(4) The trimmed reads shorter than 60 bp were discarded (--length_required 60).

The cleaned reads after being trimmed were used in the following steps.

#### Processing the Single Cell RNA Sequencing Data

We used the Seekone Tools pipeline to process the cleaned reads and generated the transcript expression matrix. First, the cell barcodes and UMI sequences were extracted based on the defined pattern about the localization of the barcode, linker, and UMI within a read. The barcode was corrected with an allow list. The corrected barcode, together with UMI, were put in the header of their corresponding reads. Second, the reads were mapped to the reference genomes using STAR 2.5.1b (Dobin et al., 2013). Then, the reads with barcode and UMI information were assigned to transcriptome using featureCounts of package Subread 1.6.4 (Liao et al., 2014) with the default parameters except “-s 1 –fracOverlap 0.5”. Finally, similar to the raw_feature_bc_matrix results of Cell Ranger (Zheng et al., 2017), the raw UMI count matrix according to barcodes and transcripts was generated. A cell-calling algorithm was used to filter the raw UMI count matrix and get the cell only filtered_feature_bc_matrix. The algorithm was similar to that of Cell Ranger and EmptyDrops (Lun et al., 2019), which had two key steps:

(1) It used a cutoff based on total UMI counts of each barcode to identify cells. This step identified the primary mode of the high RNA content cells.
(2) Then the algorithm used the RNA profile of each remaining barcode to determine if it is an “empty” or a cell containing partition. This step captured the low RNA content cells with total UMI counts similar to the empty wells.

#### Clustering and Visualization

The clustering and visualization were finished by Seurat, with the following steps:

(1) Data normalization. LogNormalize, a global-scaling normalization method, was employed to normalize the expression. The expression measurement of one transcript was divided by those of all the transcripts of the cell and multiplied by a scale factor (10,000 by default), and then the result was logarithmically transformed.
(2) Detection of highly variable features. FindVariableFeatures was used to get 2000 features per dataset.
(3) Scaling. A linear transformation (“scaling”), a standard pre-processing step prior to dimensional reduction techniques, was applied.
(4) Dimensional reduction. PCA on the scaled data was performed, and the first 15 principal components were used in the following steps.
(5) Clustering. A graph-based approach was applied to cluster the cells.
(6) tSNE/UMAP. The non-linear dimensional reduction technique was used to visualize and explore these datasets.
(7) Cluster markers. FindAllMarkers with the default parameters except “logfc.threshold = 1” was used to find markers that determined the cell clusters via the differential expression, and the top 9 markers were visualized.

### RNA-Seq

Total RNA was purified from MSCs. RNA library preparation and sequencing were performed as recommended by the manufacturer of VAHTS™ Stranded mRNA-seq Library Prep Kit for Illumina v2 (Vazyme Biotech). The library quality was assessed using Bioptic Qsep100 Analyzer. RNA-seq was performed using Illumina NovaSeq 6000 and genes with TPM value were identified afterward.

### Statistical Analysis

All results are expressed as mean ± SD. Statistical comparisons were made using a two-tailed Student’s t-test (between two groups) or a one-way analysis of variance (for multi-group comparisons). *p* < 0.05 was considered to represent a significant difference. Analysis and graphing were performed using the Prism 7.0 software package.

## Results

### Choice of Ingredients for Cocktails

**FIGURE 1:**
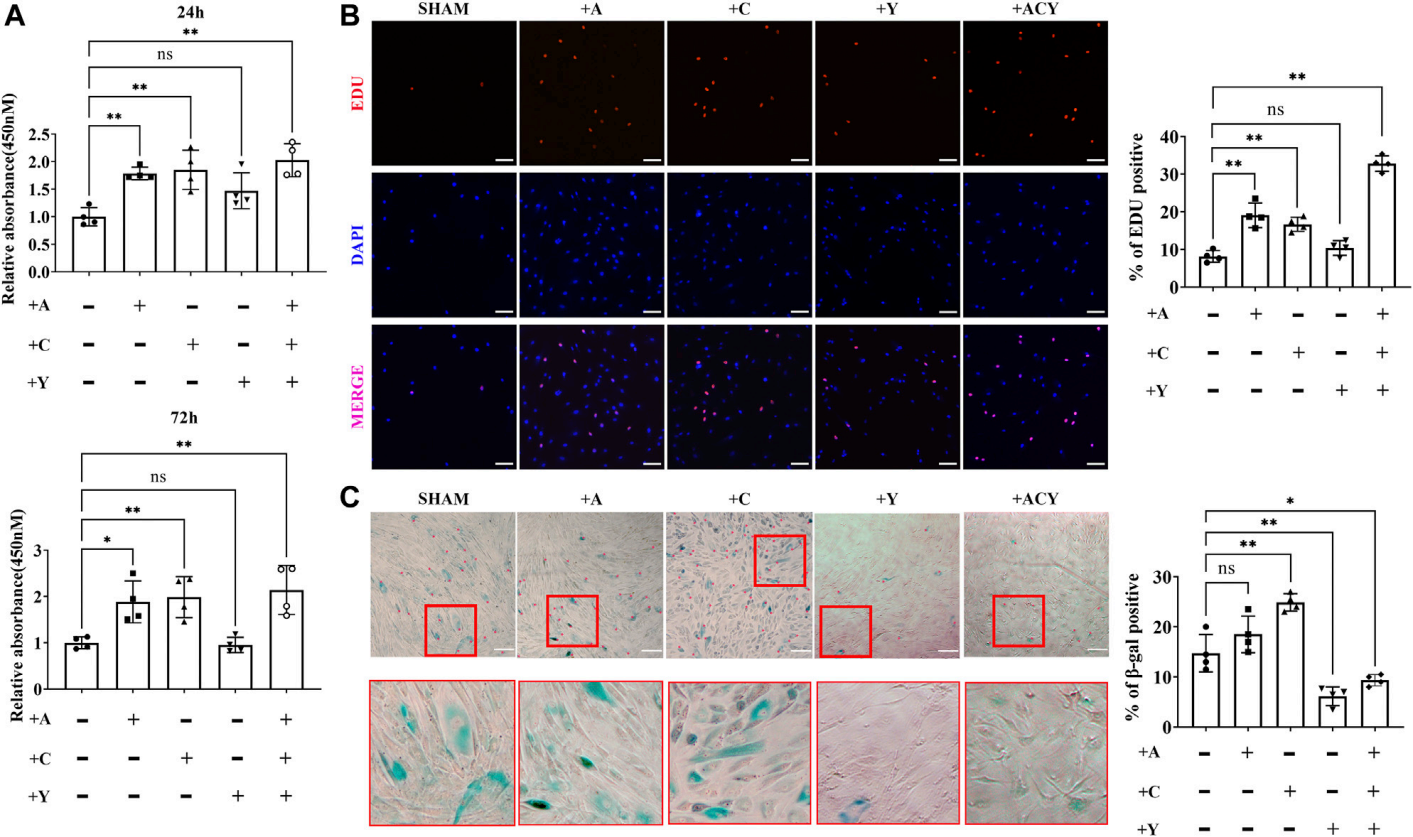
Optimization of the composition and processing time of the cocktail of small molecule compounds. **(A)** Detection of cell viability with CCK-8 assay kit in the presence of A (A-83–01), C (CHIR99021), and Y (Y27632). The values are normalized to the group without A, C, and Y, *n* = 4 per group. **(B)** Representative images of EdU staining, *n* = 4 per group. Scale bar = 100 μm. **(C)** Representative images of SA-β-gal staining, *n* = 4 per group. Scale bar = 200 μm. Data are shown as mean ± SD, *p < 0.05, **p < 0.01. and ns means not significant.

The cell counting kit-8 assay (CCK-8; Figure 1A, Supplementary Figure S2) and EDU staining (Figure 1B) indicated that A-83–01 (type 1 transforming growth factor-β receptor inhibitor, A) and CHIR99021 (glycogen synthase kinase-3 inhibitor, C) could both improve cell proliferation. Of these conditions, 0.5, 1, and 2 μM of A (Supplementary Figure S2A), or 1, 3, and 5 μM of C (Supplementary Figure S2B) could promote the proliferation of FMSCs in a time-dependent manner. However, 8 μM or higher concentrations of A and 9 μM or higher concentrations of C exhibited drug toxicity and caused cell death. Thus, 2 μM A and 3 μM C were chosen as optimal concentrations in subsequent experiments. After treatment with either A, C, or both A and C, the proliferation rate of FMSCs was upregulated within 72 h. Since cell senescence can decrease the proliferation rate, we performed the *β*-galactosidase (β-gal) staining and found that cells treated with A and especially C displayed an evident upregulation of *β*-gal-positive cell populations (Figure 1C).

Y-27632 (Rho-associated kinase inhibitor, Y), another small-molecule used to decrease apoptosis during the recovery of induced pluripotent stem cells (iPSCs), was shown to promote cell self-renewal (Gauthaman et al., 2010; Piltti et al., 2015; Wang et al., 2017). However, we found that Y could not effectively promote cell proliferation alone, and 10 μM or higher concentrations of Y inhibited cell viability (Figure 1A and Supplementary Figure S2C). Thus, less than 10 μM Y was considered as a concentration that does not significantly decrease cell proliferation rate, and 2 μM was chosen as the optimal concentration in the subsequent experiments. Interestingly, the addition of Y can significantly increase the cell proliferation rate based on A and C (Figures 1A,B), and the addition of Y could significantly downregulate cell senescence (Figure 1C). Thus, we identified the ACY small-molecule cocktail system composed of three small molecules, and further investigated the improvement effect on MSCs.

### Proliferation

**FIGURE 2:**
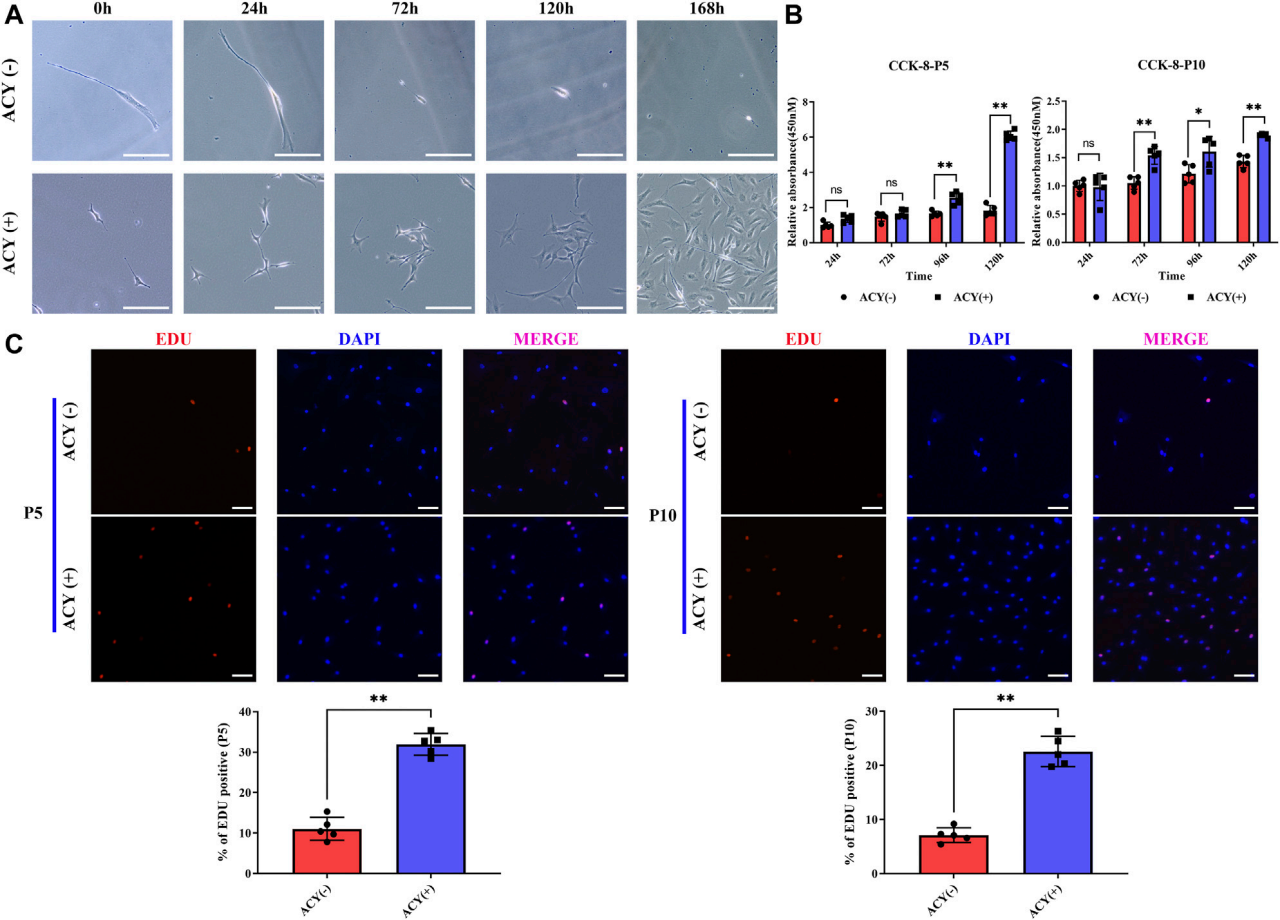
ACY promotes the cell proliferation of MSCs. **(A)** The ability of MSCs to form clones under exceptionally low seeding density in 168 h. Scale bar = 200 μm. **(B)** Detection of cell viability at each passage with CCK-8 assay kit. The values are normalized to the ACY (-) group of 24 h, *n* = 5 per group. **(C)** Representative images of EdU staining at each passage, *n* = 5 per group. Scale bar = 100 μm. The values are normalized to the ACY (-) group. Data are shown as mean ± SD, *p < 0.05, **p < 0.01, and ns means not significant.

We compared the proliferation effects in cultures with DMEM media containing FBS or DMEM containing FBS and the ACY cocktails. Cell passaging was conducted at low density to individually trace the viability and clone formation ability of sparsely inoculated FMSCs. Without ACY stimulation, proliferating cells were rarely observed, and many cells died (Figure 2A). In the presence of ACY, cell viability and clone formation ability of FMSCs was improved (Figure 2A). The CCK-8 assay (Figure 2B) and EDU staining (Figure 2C) demonstrated a clear difference in cell viability and proliferation rates between the ACY (-) and ACY (+) conditions. Additionally, cells in the ACY (+) group needed less time to expend to a passaging density (Supplementary Figures S3A–B).

When FMSCs were expanded in ACY-supplemented medium, their expression profiles became distinct (Supplementary Figure S4A). In total, 1909 genes were downregulated and 1691 were upregulated in ACY medium-expanded hMSCs from P5 and P10 (Supplementary Figures S4B,C). In the presence of ACY, proliferation genes, such as cell cycle-related genes (CCNA2, CCNB1, CCNB2, CDK1, CDK2, CDK6, CENPE, CENPF, MCM2, MCM3, MCM4, MCM5, MCM6, MKI67, and PCNA), were positively upregulated (Supplementary Figure S4D).

To validate the reliability and reproducibility of ACY stimulation, we determined whether ACY could be used for other types of MSCs. The same tests were applied to bone marrow-derived MSCs (BMSCs) and umbilical cord blood-derived MSCs (UMSCs). CCK-8 assay (Supplementary Figure S5A) and EDU staining (Supplementary Figure S5B) implied that ACY upregulates the proliferation rate of these two types of MSCs in a time-dependent manner.

The results presented here suggest that ACY effectively promotes MSCs proliferation.

### Senescence Analysis

**FIGURE 3:**
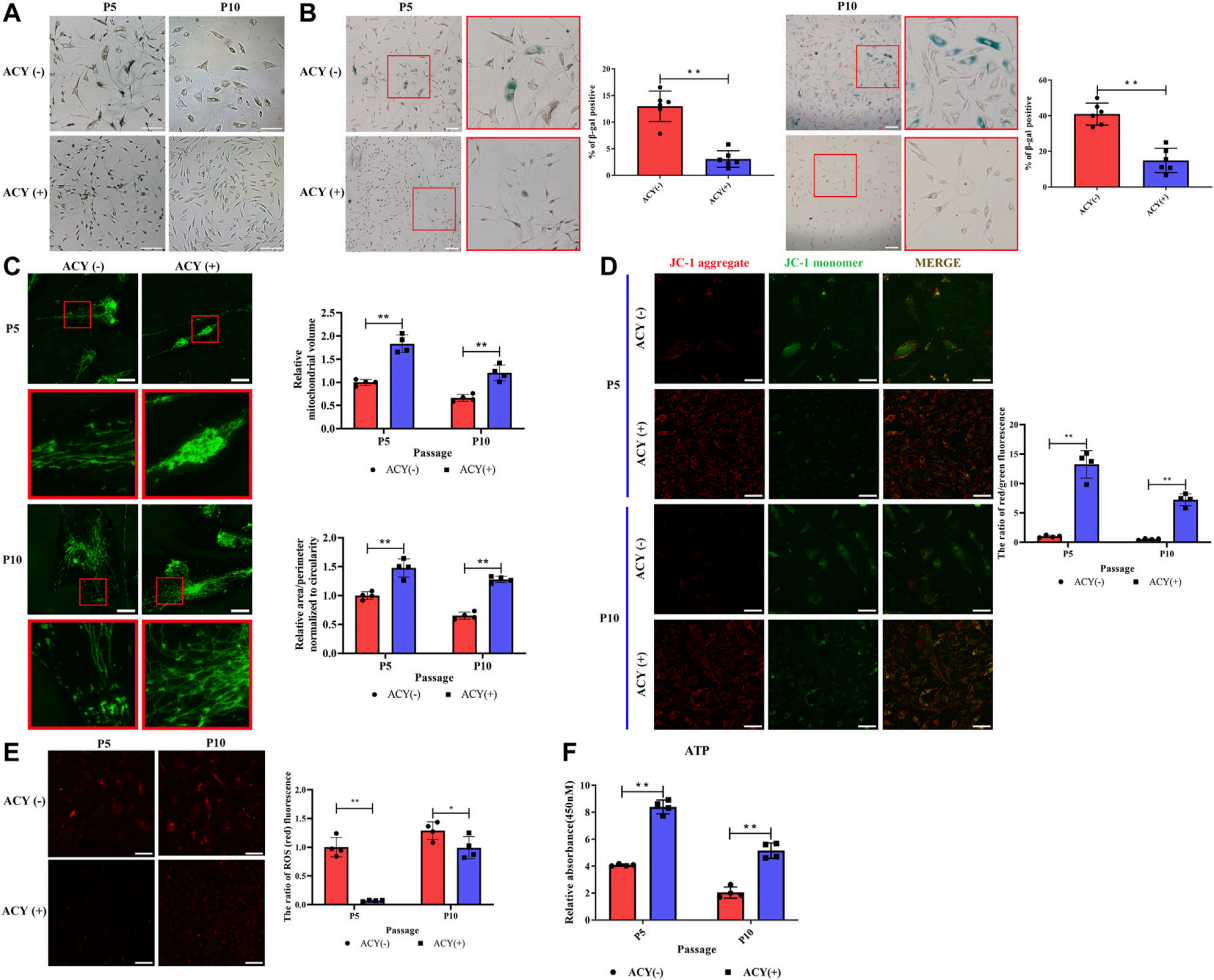
Comparison of senescence of MSCs cultured in ACY (-) and ACY (+) conditions. **(A)** Representative images of MSCs cultured with or without ACY at each passage. Scale bar = 100 μm. **(B)** Representative images of SA-β-gal staining at each passage, *n* = 6 per group. Scale bar = 100 μm. **(C)** Representative micrographs of MitoTracker-labeled MSCs in the presence of ACY (-) and ACY (+) conditions at each passage, the mitochondrial volume and the mean (area/perimeter)/circularity index are normalized to the ACY (-) group, *n* = 4 per group. Scale bar = 25 μm. **(D)** MSCs of ACY (-) and ACY (+) groups at each passage were stained by JC-1, *n* = 4 per group. Scale bar = 40 μm. The relative fluorescence intensity of JC-1 was calculated. The mean fluorescence intensity values in the ACY (-) group of P5 were normalized to 1.0. **(E)** Representative images of ROS staining at each passage, *n* = 4 per group. Scale bar = 75 μm. The relative fluorescence intensity values in the ACY (-) group of P5 were normalized to 1.0. **(F)** Detection of ATP generation at each passage, *n* = 4 per group. The relative RLU values in the ACY (-) group of P5 were normalized to 1.0. Data are shown as mean ± SD, *p < 0.05, **p < 0.01, and ns means not significant.

Our data indicated that FMSCs treated with ACY had a thinner and more normal fusiform and spindle-shaped morphology in P5 and P10 (Figure 3A). The effects of ACY on cellular senescence were examined with *β*-gal staining; ACY significantly reduced the rate of *β*-gal-positive cells (Figure 3B). In the presence of ACY, the rate of *β*-gal-positive cells was also downregulated in BMSCs (Supplementary Figure S6A) and UMSCs (Supplementary Figure S6B).

Mitochondria activity is closely related to the process of cell senescence (Wang et al., 2020; Zhao et al., 2021); therefore, we examined activity with a series of analyses. First, we examined the morphology of mitochondria in the ACY (-) and ACY (+) conditions. MitoTracker staining showed a more fragmented morphology of mitochondria in MSCs without ACY (Figure 3C). JC-1 staining was carried out to detect the mitochondrial membrane potential, and the results showed that ACY treatment induced an evident increase in red fluorescence intensity. This suggests that ACY upregulates the mitochondrial membrane potential of MSCs (Figure 3D). Levels of reactive oxygen species (ROS) in cells are one of the key indicators for measuring mitochondrial activity. With the aggravation of cell senescence and the decline of mitochondrial function, the level of ROS increases gradually. Our data indicated that ACY could effectively limit the upregulation of ROS levels with an increase in cell generation (Figure 3E). Furthermore, the intracellular ATP levels in the ACY (+) condition were significantly higher than that of the ACY (-) group (Figure 3F).

Expression profiling indicated that among the differentially expressed genes in MSCs, the groups (MDM2, COL4A2, and BCL11A) known to induce senescence were downregulated, whereas the genes known to drive cell cycling and prevent aging (LMNA, SPRY2, STIP1, MAPKAPK5, and FANCD2) were upregulated (Supplementary Figure S4E).

Collectively, these data show that ACY could effectively reduce MSCs senescence.

### Differentiation Analysis

MSCs are multipotent and can differentiate into three separate mesenchymal lineages. The mRNA expression of transcripts typical to fat, bone, and cartilage lineages was affected by ACY supplementation. Peroxisome proliferator activated receptor gamma (PPARG), pro-brown adipose tissue gene FOXC2 and anti-white adipose tissue gene GATA2 were upregulated in an ACY (+) group of MSCs (Supplementary Figure S4F). Osteogenesis-related genes were highly expressed in the ACY (+) group, including the regulator of osteogenesis gene COL11A1, the predictor of heterotopic ossification TGFβ3, and the transcription factor strongly associated with the osteoblast phenotype called RUNX2 (Supplementary Figure S4F). Additionally, five genes related to chondrogenesis were also upregulated in the ACY (+) group, including COL1A1, PRRX2, NFIB, OSR1, and SFRP2 (Supplementary Figure S4F).

**FIGURE 4:**
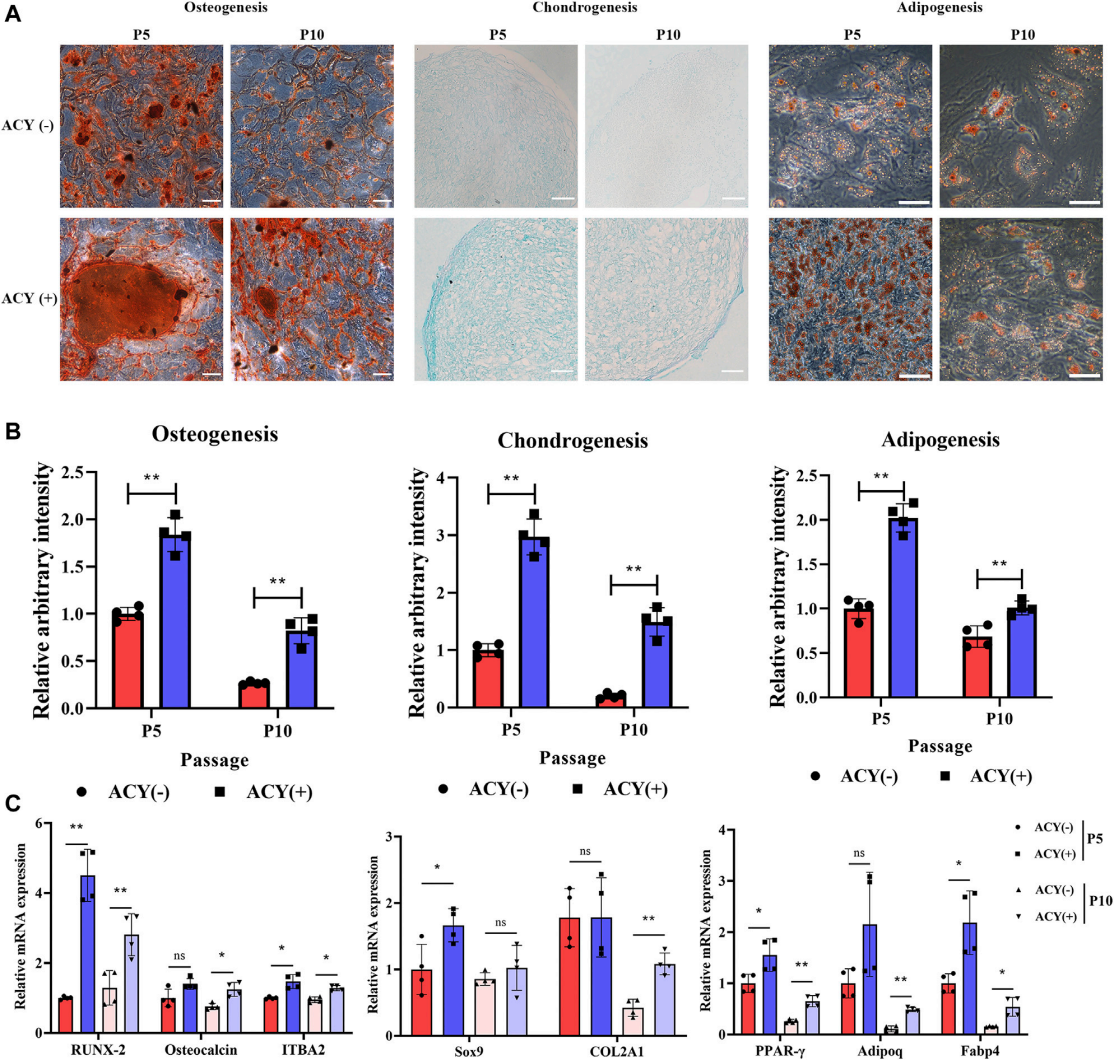
Multilineage differentiation potential of MSCs. **(A)** Differentiation into osteocytes, chondrocytes, and adipocytes was induced and stained. Scale bar = 100 μm. **(B)** The staining intensity was quantitated and evaluated by ImageJ. The values are normalized to the ACY (-) group of P5, *n* = 4 per group. **(C)** Multilineage differentiation potentials of osteogenesis, chondrogenesis, and adipogenesis were detected by qPCR, *n* = 4 per group. Data are shown as mean ± SD, *p < 0.05, **p < 0.01. and ns means not significant.

To further examine whether differentiation could be affected by ACY conditions, MSCs cultured with or without the ACY cocktail were examined in a specific medium used to initiate differentiation into one of three lineages. Each sample set was observed after 21 days of differentiation. The osteogenic differentiation of MSCs in the ACY (-) and ACY (+) groups was observed by alizarin red staining of calcium deposits. Adipogenic differentiation was observed with Oil Red O staining of lipid droplets. Chondrogenic differentiation was observed with Alcian blue staining of the internal acid mucopolysaccharide. Our data indicated that both ACY (-) and ACY (+) groups showed trilineage differentiation capabilities, and differentiation potentials decreased from P5 to P10 (Figure 4A). Densitometric analysis showed that the characteristic staining of osteogenic, chondrogenic, and adipogenic differentiation of MSCs cultured in ACY (+) conditions were more evident than those cultured in ACY (-) conditions (Figure 4B). Furthermore, the ACY (+) group exhibited upregulation of mRNA expression of osteogenic (RUNX-2, *osteocalcin,* and ITBA2), chondrogenic (SOX9 and COL2A1), and adipogenic (PPARG, ADIPOQ, and FABP4) factors (Figure 4C).

These results indicated that ACY effectively promotes the stem cell potential of MSCs.

### Cell Homogeneity Analysis

**FIGURE 5:**
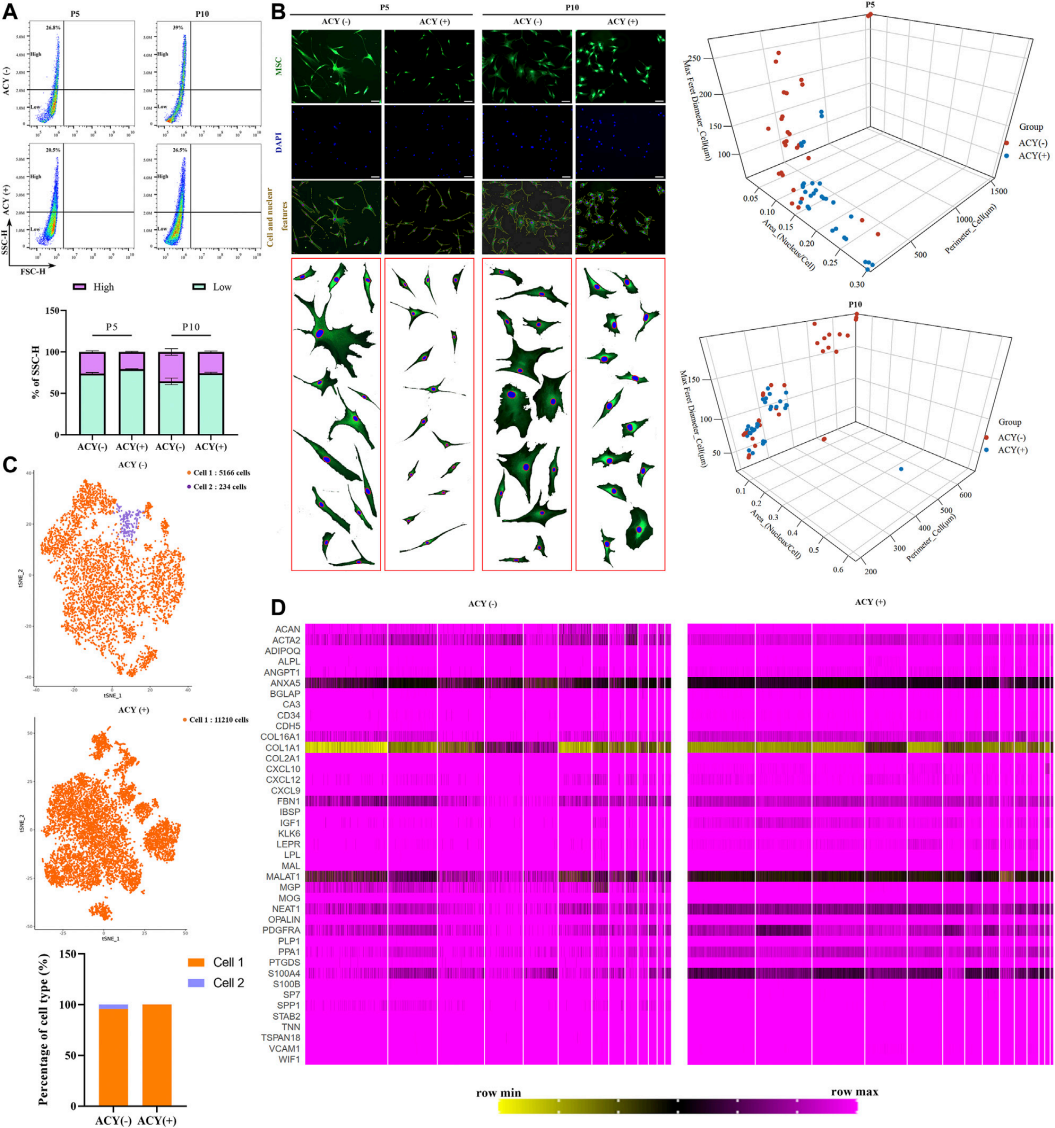
Comparison of cell homogeneity. **(A)** Cellular homogeneity of MSCs was analyzed by FACS, *n* = 4 per group. Data are shown as mean ± SD. **(B)** Three-dimensional graphs showing correlation of the morphological features (perimeter, nucleus/cytoplasm ratio, maximum Feret diameter) using morphological data from MSCs in ACY (-) and ACY (+) conditions. Data in ACY (-) and ACY (+) represent morphological data from three independent experiments (*n* = 30 total points). Scale bar = 75 μm. **(C)** Comparison of the heterogeneity of MSCs by single-cell sequencing and cluster analysis. **(D)** Comparison of the gene expression in MSCs by single-cell sequencing. A total of 41 genes about osteoblasts, chondrocytes, Schwann cells, and vascular cells were analyzed.

The side scatter channel (SSC) is a measure of the cell refractive index, which is dependent on the cell granularity or internal complexity (Ramirez et al., 2013; Lee et al., 2017). It is known that a high SSC population implies increased apoptosis and aneuploidy compared to that in the low SSC population [36]. Our data showed that MSCs cultured in the ACY (-) condition were more heterogeneous compared to those in the ACY (+) condition, and SSC values increased with consecutive cultures across passages in the ACY (-) group, whereas the ACY (+) group maintained a relatively stable SSC population (Figure 5A).

Cells in both ACY (-) and ACY (+) groups exhibited fibroblastic morphology, but MSCs cultured in the ACY (-) condition showed higher heterogeneity. The ACY (-) group showed a lower nucleus to cell area ratio, a wider diameter and larger adherent sizes, and the morphologies across passages showed increased variation (Figure 5B). The ACY (+) group maintained a fibroblastic morphology with a slight cell body enlargement and improved homogeneity. To examine the heterogeneity features, MSCs in the ACY (-) and ACY (+) groups were subjected to single-cell RNA sequencing. Our data showed that all MSCs in the ACY (+) group could be identified as the same cell type, but 234 cells in 5400 MSCs in the ACY (-) group were identified as a different cell type (Figure 5C). These results indicate that ACY effectively promoted the homogeneity of MSCs.

To further test whether ACY could promote the homogeneity of MSCs, we analyzed the single-cell RNA sequencing data and compared the gene expression profiles. Based on the markers reported to be expressed by MSCs and other cell types with similar phenotypes, such as osteoblasts, chondrocytes, Schwann cells, and vascular cells (Baryawno et al., 2019; Tikhonova et al., 2019; Leimkuhler et al., 2021), we compared expression levels in the ACY (-) and ACY (+) groups. Although there was no evident difference in the expression of the markers mentioned above, MSCs in the ACY (+) group showed a smaller difference in the individual gene expression levels, such as ACAN, ACTA2, ANXA5, COL16A1, COL1A1, FBN1, MALAT1, NEAT1, PPA1, S100A4, and SPP1(Figure 5D, Supplementary Figures S7A–B).

Additionally, karyotype analysis confirmed a normal karyotype in MSCs after long-term treatment with ACY (Supplementary Figure S8A). Telomerase activity is a widespread and relatively selective tumor cell marker. We tested the telomerase activity of MSCs, and our results confirmed that MSCs of the ACY (+) group showed a slightly elevated telomerase activity compared to the ACY (-) group. However, the increasing tendency gradually weakened with increases in cell passage, and telomerase activity did not increase significantly in MSCs of the ACY (+) group (Supplementary Figure S8B).

Overall, ACY did not alter the phenotype of MSCs, but was able to improve gene stability.

### Surface Marker and Functional Cytokines Expression

**FIGURE 6:**
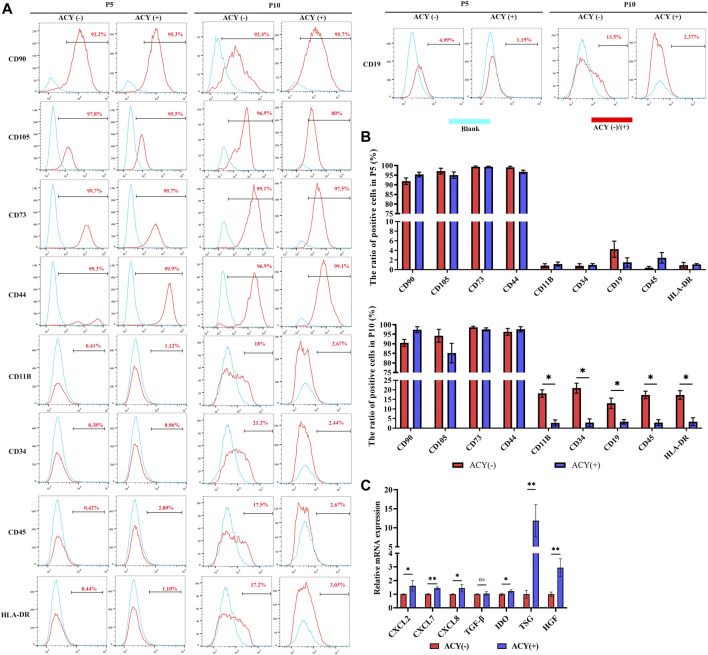
Comparison of cell phenotype. **(A** ,**B)** Flow cytometry was adopted to analyze the expression of surface markers. *n* = 3 per group. **(C)** The mRNA levels of chemokines and secreted factors. *n* = 4 per group. Data are shown as mean ± SD, *p < 0.05, **p < 0.01, and ns means not significant.

Flow cytometry analysis showed that the FMSCs in the ACY (-) and ACY (+) groups exhibited a conventional MSC surface marker profile. Both groups expressed high levels of CD90, CD105, CD44, and CD73, but lacked expression of CD11B, CD19, CD34, CD45, and HLA-DR surface molecules at P5 (Figures 6A,B, Supplementary Figure S4G). However, as the cell generation increased to P10, the expression of negative markers such as CD34, CD11B, HLA-DRs, CD45, and CD19 was slightly upregulated in the ACY (-) FMSC group (Figures 6A,B, Supplementary Figure S2G). To test the expression of some classic chemokines and paracrine factors in MSCs, cells were treated with TNF-α and IL-1β for 24 h to stimulate expression. Our results showed that MSCs from the ACY (+) group showed a more evident upregulation of most functional genes (CXCL2, CXCL7, CXCL8, IDO, TSG, and HGF) than the ACY (-) group (Figure 6C).

## Discussion

Here, we provide a simple and effective method for enhancing the growth of MSCs. We combined three small-molecule compounds A-83–01, CHIR99021, and Y27632 in a defined proportion and added the ACY cocktail to the culture medium during MSC expansion. A-83–01 is an inhibitor of the TGF-β signaling pathway. Many reports suggest that TGF-β stimulation can promote the proliferation and prevent apoptosis of MSCs (Stewart et al., 2010; Walenda et al., 2013; Gurung et al., 2020), and other studies have shown that activation of the TGF-β pathway can cause cell senescence (Ito et al., 2007; Gurung et al., 2020). CHIR99021 is an activator of the Wnt signaling pathway. Activation of the Wnt pathway is believed to strongly induce MSC proliferation (Boland et al., 2004; Jothimani et al., 2020). However, activation of the Wnt pathway is also believed to cause MSC senescence by promoting the production of ROS and activating p53/p21 (Gu et al., 2014; D. Y.; Zhang et al., 2013). Y-27632 is an inhibitor of the ROCK signaling pathway, known to promote the proliferation of MSCs (Nakamura et al., 2014). Although several studies have used a combination of A-83–01, CHIR99021, and Y27632 to regulate the function of specific stem cells, such as the conversion of terminally committed hepatocytes to culturable bipotent progenitor cells, our study demonstrated that ACY can promote proliferation and maintain gene stability and homogeneity of MSCs, which can be clearly distinguished from these previous reports.

The results presented here show that ACY can reduce MSC senescence. Numerous studies reported that expanding MSCs in the nutrient-rich artificial culture environment *in vitro* for rapid proliferation could reconfigure their central energy metabolism (Pattappa et al., 2011; Sherr and DePinho 2000; Yuan et al., 2019a), which results in accumulation of ROS and further leads to mitochondrial dysfunction, energy failure, and metabolic disorders (Babizhayev and Yegorov 2015). Loss of mitochondrial function can drive a senescence growth arrest (Wiley et al., 2016), while at the same time cell senescence directly leads to senescence-associated mitochondrial dysfunction (Passos et al., 2010). In functional mitochondria, generation of ROS, membrane potential, and ATP production are tightly regulated (Brand 2016), whereas a decreased membrane potential and increased levels of ROS occur in dysfunctional mitochondria of senescence cells. Maintaining normal mitochondrial function is of great significance to inhibiting cell aging. Relative signaling pathways are known to be associated with mitochondrial fitness regulation. TGF-β signaling pathway can induce fragmented mitochondria and trigger apoptosis (Gao et al., 2020). Wnt/β-catenin signaling pathway can induce mitochondrial biogenesis (Undi et al., 2017; Yoon et al., 2010), and inhibit ROCK signaling pathway which may attenuate mitochondrial fragmentation (Liu et al., 2019). In this work, we show that ACY can maintain mitochondrial fitness while promoting rapid MSC expansion, which is consistent with previous results mentioned above.

MSCs need to be expanded *in vitro* for a period of time before they can be used in cell therapy. However, continuous subculture results in senescence, which manifests as morphological abnormalities (Bonab et al., 2006; Yang et al., 2018), cell enlargement (Majd et al., 2009; McCorry et al., 2016), and proliferation arrest (Alessio et al., 2013; Melzer et al., 2020). Proliferation, senescence, and differentiation potential are three issues that challenge the expansion of MSCs. In fact, MSCs have been shown to lose multipotency after prolonged passaging. We found that by adding the ACY cocktail with a specific composition ratio to the culture system of MSCs, these three characteristics can be significantly improved. As cell viability improved, it was not surprising that the multipotency of MSCs was also improved. However, TGF-β, Wnt, and ROCK signaling pathways are involved in coordinating the differentiation process. For example, adipose tissue can be divided into white and brown adipose, and TGF-β can inhibit both brown and white preadipocyte formation (Grafe et al., 2018). Further, activated Wnt–β-catenin signaling increases osteoblast differentiation and likely contributes to the chondrocyte differentiation phenotype (Rawadi et al., 2003). ROCK inhibitors lead to increased expression of stem cell markers, which help to maintain multipotent MSCs *in vitro* (Zhang and Kilian 2013). Therefore, each compound impacts a specific pathway to modulate developmental potential, and together improve multipotency in MSCs.

Recently, an increasing number of studies have reported that MSCs show evident heterogeneity which contributes to unpredicted cell population dynamics and functional alterations after extended culture (Russell et al., 2010; Samsonraj et al., 2015). MSCs include no more than three morphologically different cell populations: (1) small and rapidly proliferating cells, (2) fibroblast-like cells, and (3) large, flat, slowly self-renewing cells (Colter et al., 2001; Prockop et al., 2001; Costa et al., 2021). Cell heterogeneity may partially explain the incongruent data from the MSC-based clinical trials (Phinney 2012). During MSC passaging and large-scale expansion *in vitro*, the subpopulations of MSCs may change, resulting in cell function alterations (Sethe et al., 2006; Bustos et al., 2014; Sun et al., 2020). Therefore, our study implies that ACY could help to maintain a relatively homogeneous culture of MSCs, which may contribute to a stable treatment outcome. HLA-DR belongs to the major histocompatibility complex class II and plays a crucial role in host immune responses. Previous reports suggest that HLA-DR could change the karyotypes and cause earlier senescence of MSCs (Dam et al., 2021). Additionally, HLA-DR-expressing MSCs may increase the risk of an alloimmune response (Kot et al., 2019). We observed that ACY stimulation reduced the expression of HLA-DR and caused no abnormality in chromosomes and telomerase activity.

MSCs possess immunomodulatory properties mediated via soluble immunomodulatory factors for a considerable extent (English 2013). Several cytokines such as HGF, IDO, TSG, and TGF-β have been shown to play an important role in immunomodulation (Ben-Ami et al., 2011). Although some culture conditions during *in vitro* expansion can significantly promote MSC proliferation, they alter the fitness of MSCs, and reduce their immunomodulatory capacity and therapeutic potency (Yuan et al., 2019). The immunomodulatory effect of MSCs depends on local immunological conditions, where inflammatory factors such as IFN-γ and TNF-α are present. Our study showed a significant upregulation of immunomodulatory factors IDO, HGF, and TSG under inflammatory cytokines’ stimulation. The results, to some extent, indicate the potential of ACY in the clinical treatment of MSCs.

Several limitations are inherent in the present study. First, we did not define the exact underlying mechanism to explain how ACY improves the quality of MSCs. As each compound of the ACY cocktail could impact a major signaling pathway, we hypothesized that their function did not focus on a critical gene or pathway, but rather on the synergy of the combined signaling pathway. Based on the current sequencing results, our future research may be able to find a new functional molecule to explain the changes that occur in MSC cultures. Second, we tested effects on cell viability, multipotentiality, homogeneity, and phenotypical characteristics of MSCs, but we have not yet tested the optimized ACY cocktail treatment of MSCs on a specific clinical model. As the classic therapeutic functions of MSCs include tissue repair, immune regulation, and hematopoietic support, we are currently investigating the most suitable disease model for ACY-MSCs. The method described here represents a significant step toward this goal and is applicable to MSCs from different tissues. Most importantly, it provides a simple and cost-effective method to improve MSC production. A defined combination of ACY in conjunction with cell culture automation may eventually lead to large-scale production of high-quality MSCs for industrial and clinical translation.

## Conclusion

**FIGURE 7:**
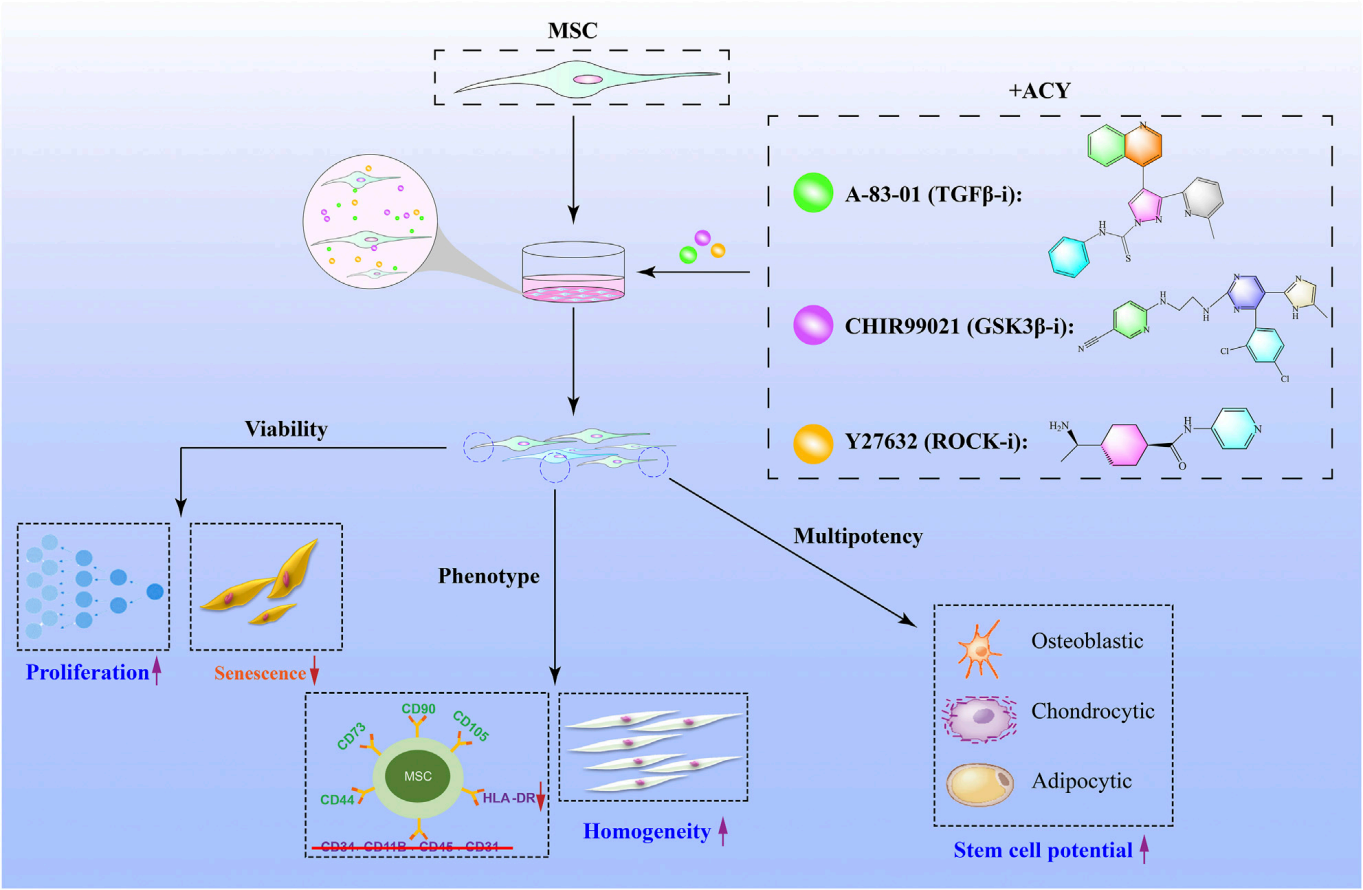
Schematic illustration of small-molecule cocktails ACY-mediated enhanced cell growth of MSCs. ACY contributes to enhance cell viability and maintain stem cell differentiation, cell homogeneity potential, and phenotype.

Our results demonstrate that the addition of ACY could be a potential strategy for the large-scale *in vitro* expansion of high-quality MSCs (Figure 7). ACY can promote MSC proliferation, inhibit senescence, and maintain cell phenotype (including genomic stability, stem cell potential, and homogeneity). In addition, ACY can prevent the upregulation of HLA-DR and promote the secretion of immunomodulatory cytokines and chemokines, demonstrating its potential for clinical application.

## Data Availability

The datasets presented in this study can be found in online repositories. The names of the repository/repositories and accession number(s) can be found below: CNCB National Genomics Data Center, accession no: PRJCA008019.
