# Supplementary material for: A Small-Molecule Cocktails-Based Strategy in Culture of Mesenchymal Stem Cells
Source: Front Bioeng Biotechnol. 2022 Mar 14;10:819148. doi: 10.3389/fbioe.2022.819148 (PMC8963903; doi:10.3389/fbioe.2022.819148)
Supplement: Supplementary file 1 [file DataSheet1.docx]

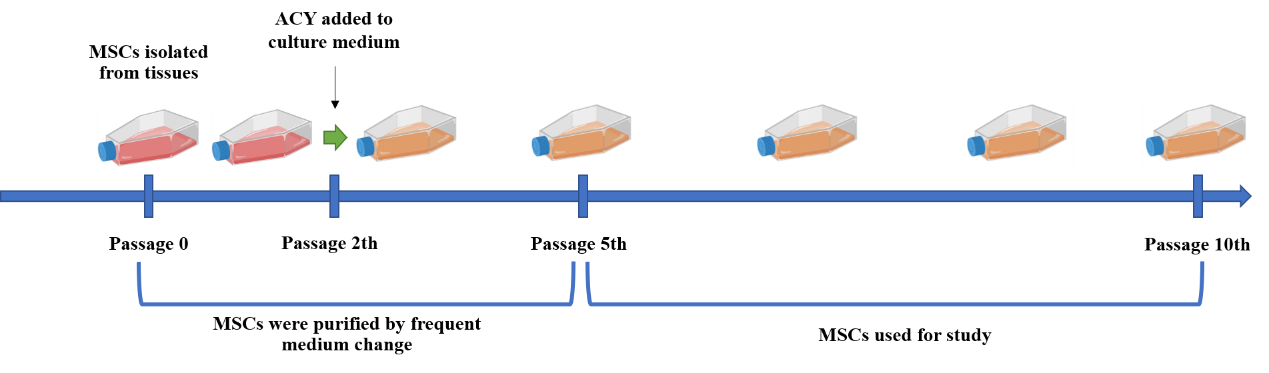


**Figure S1. Schematic diagram of the strategy to stimulate MSCs with ACY.**


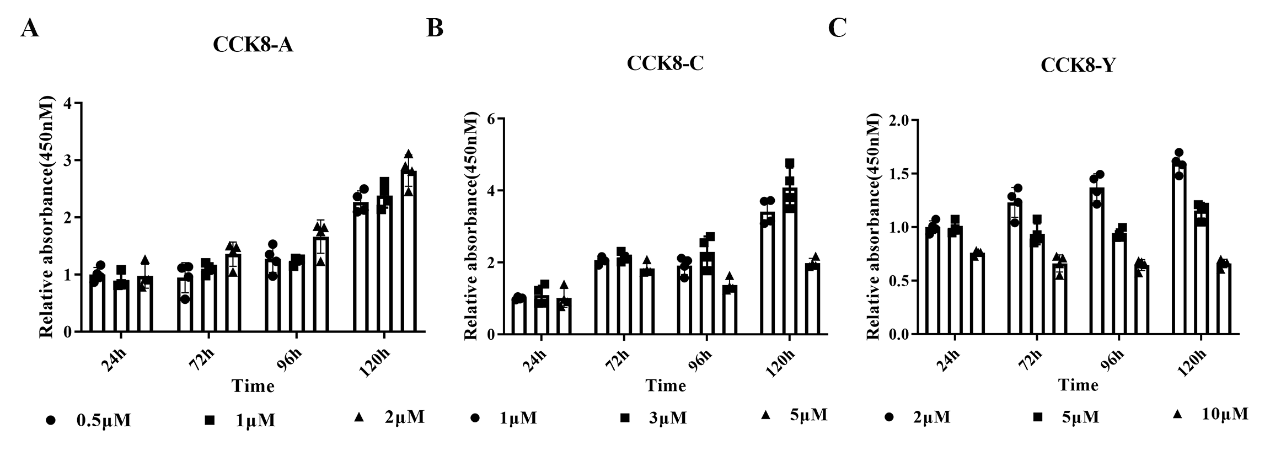


**Figure S2. Optimization of the concentration and duration of the small molecule treatment.** Detection of cell viability with CCK-8 assay kit in the presence of A (A-83-01, A), C (CHIR99021, B) and Y (Y27632, C) at 24h, 72h, 96h and 120h, n = 4 per group.


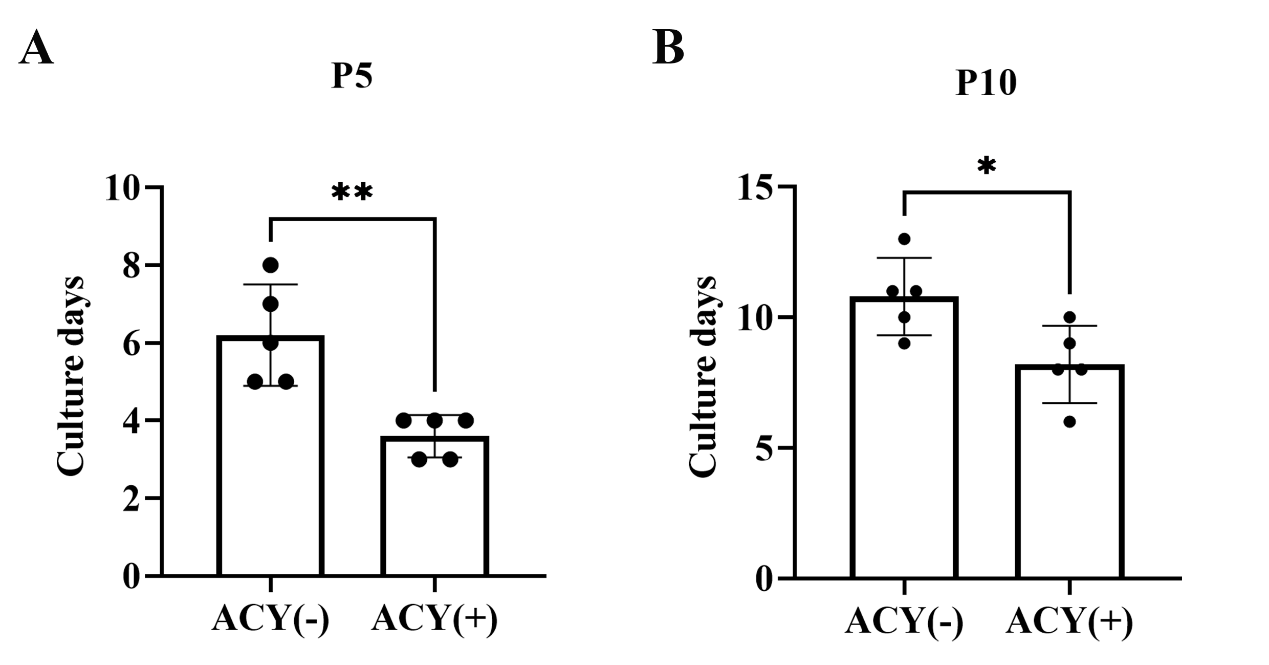


**Figure S3. Detection of MSC culture time.** MSCs (1 × 10^4^ cells/cm^2^) were cultured in T25 cell culture flasks. The time was measured when cells of P5 (A) and P10 (B) were approximately 90% confluent. n = 5 per group. Data are shown as mean ± SD, *P < 0.05 and **P < 0.01.


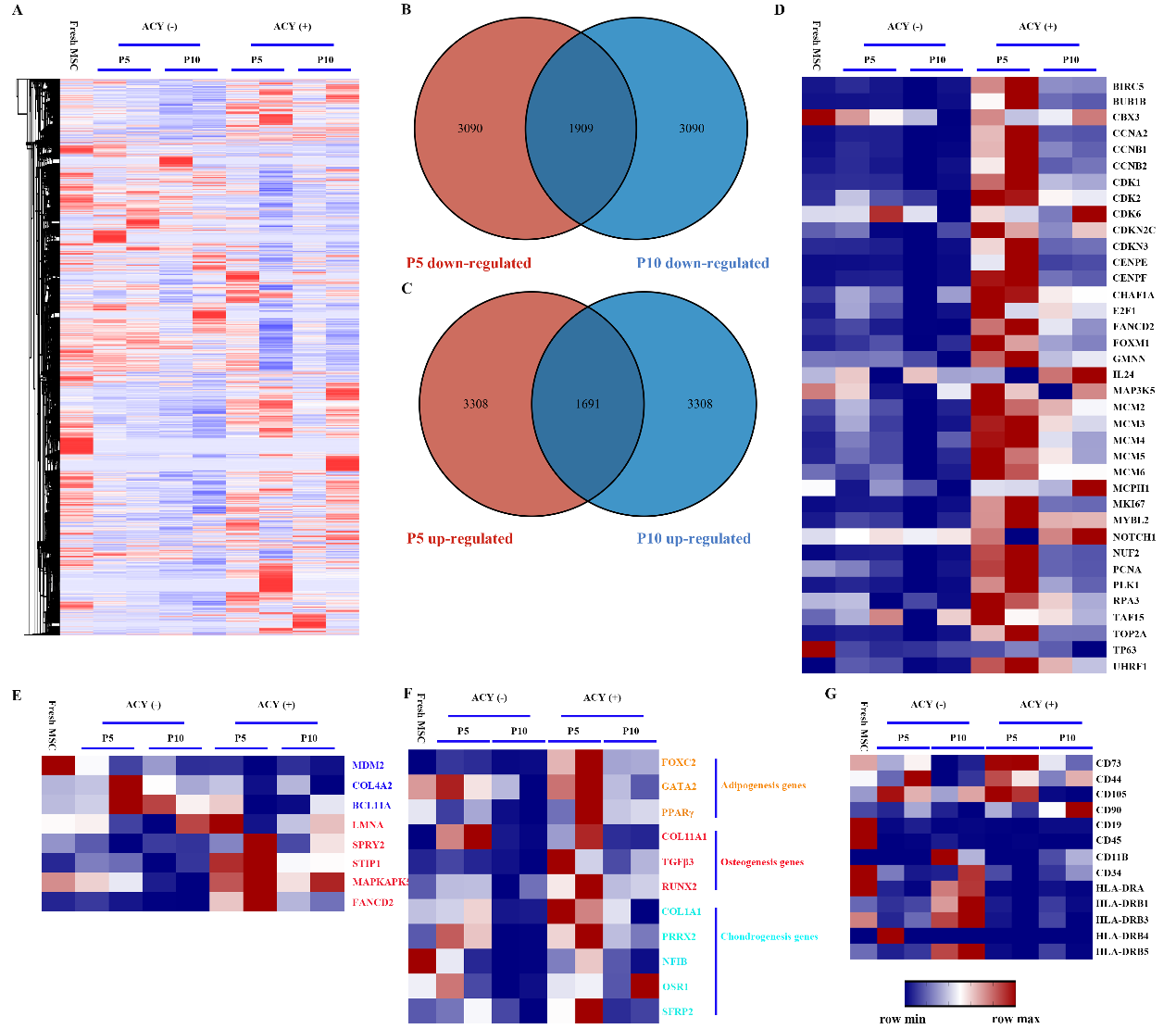


**Figure S4. RNA-seq analyses of MSC**. (A)Hierarchical clustering of differentially expressed genes on FMSCs; The Venn Diagram to show numbers of genes differentially expressed in FMSCs of P5(B) and P10(C); Freshly isolated MSCs and cells cultured for the designated periods with or without ACY stimulation were used for RNA-seq analyses. Genes that were differentially expressed between the ACY (-) and YAC (+) conditions on P5 and P10 were selected. Of these, 37 cell proliferation-related genes were manually picked up(D). RNA-seq analyses of cell senescence-related genes(E). Multilineage differentiation-related genes (F) and surface markers(G) were picked up.


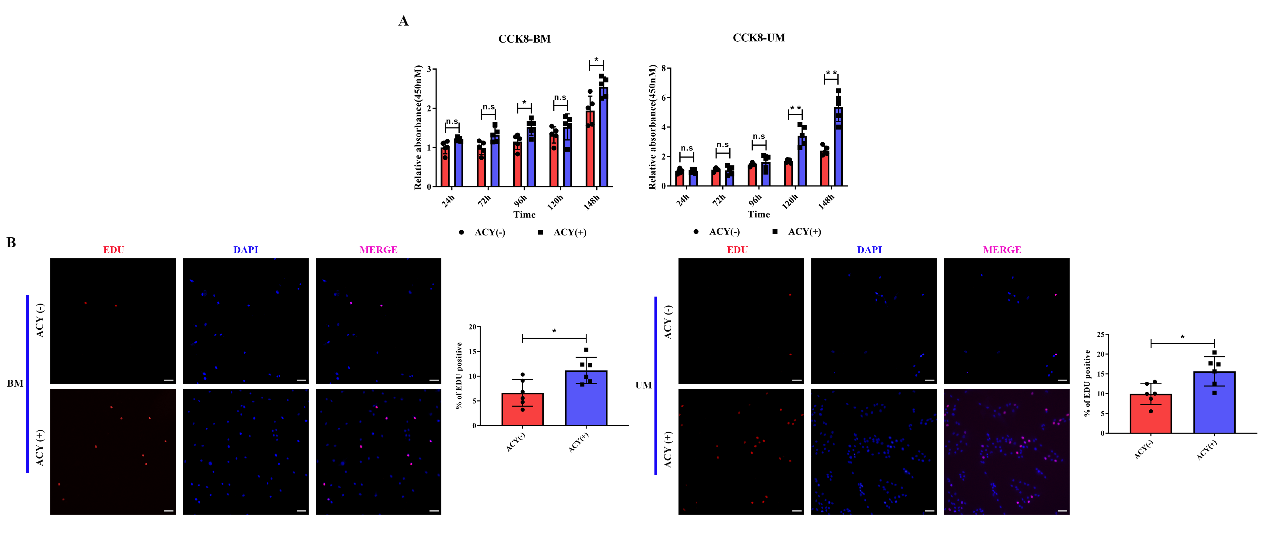


**Figure S5. ACY promote the cell proliferation of BMSCs and UMSCs.** (A) Detection of cell viability at each passage with CCK-8 assay kit. The values are normalized to the ACY (-) group of 24h, n = 5 per group. (B) Representative images of EdU staining at each passage, n = 5 per group. Scale bar = 100μm. Data are shown as mean ± SD, *P < 0.05, **P < 0.01.


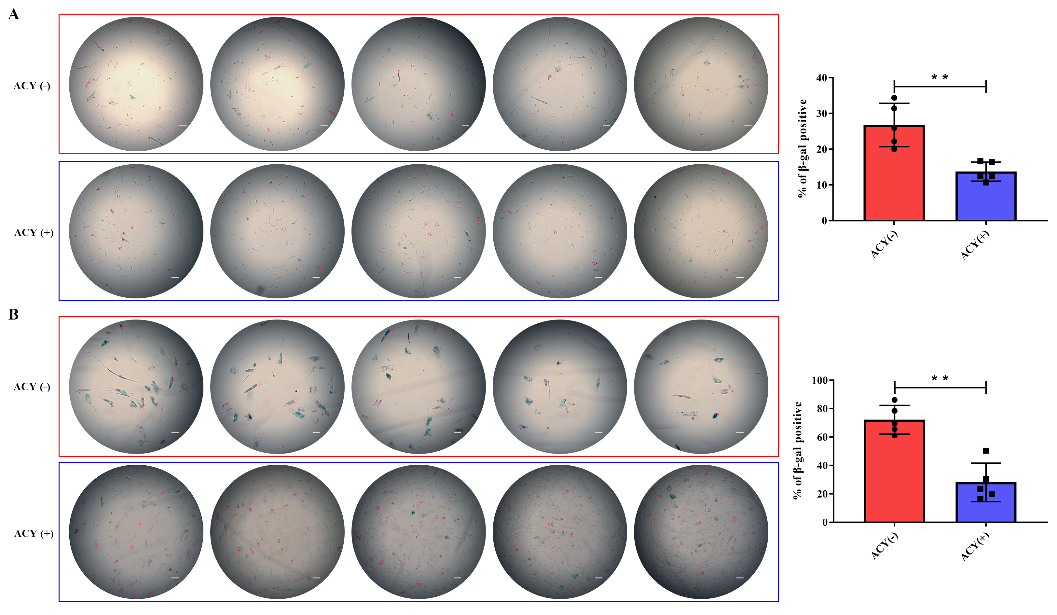


**Figure S6. Comparison of senescence of BMSCs and UMSCs cultured in ACY (-) and ACY (+) conditions.** Representative images of SA-β-gal staining of BMSCs (A) and UMSCs (B), n = 5 per group. Scale bar = 100μm. Data are shown as mean ± SD. **P < 0.01.


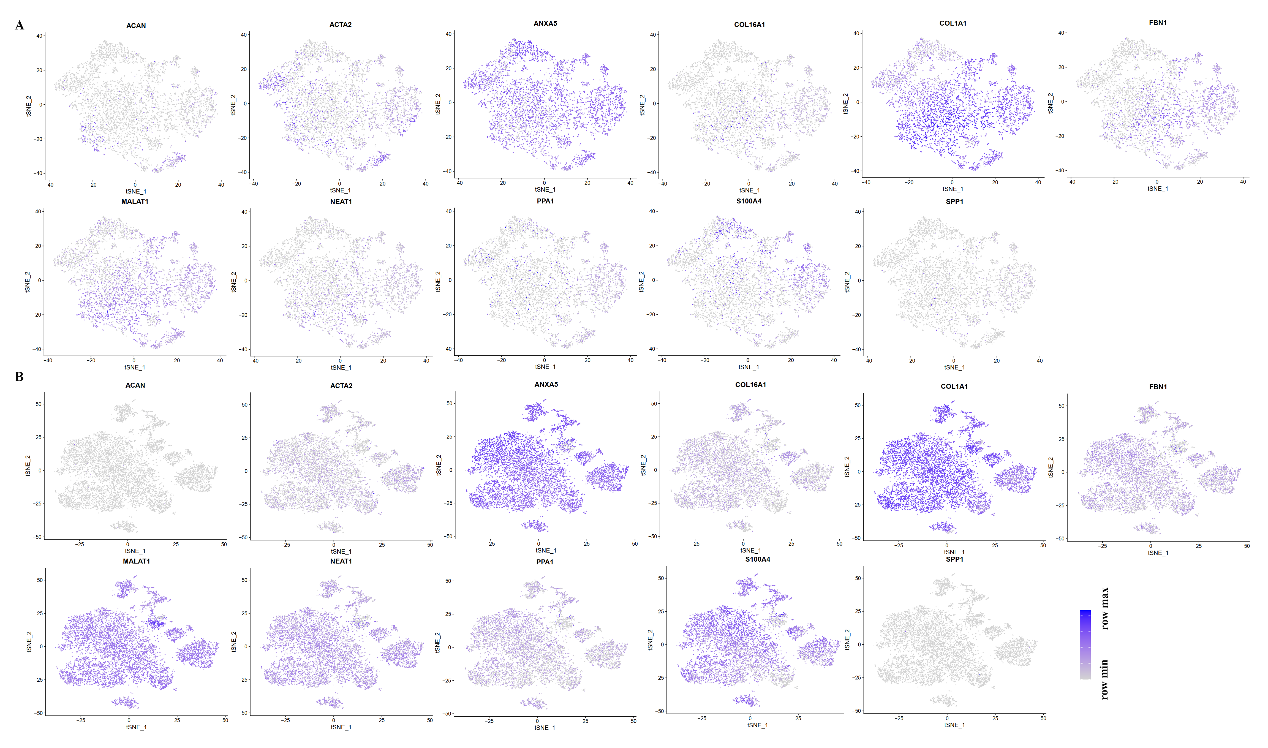


**Figure S7. Comparison of the gene expression in MSCs without (A) or with (B) ACY by single-cell sequencing**.


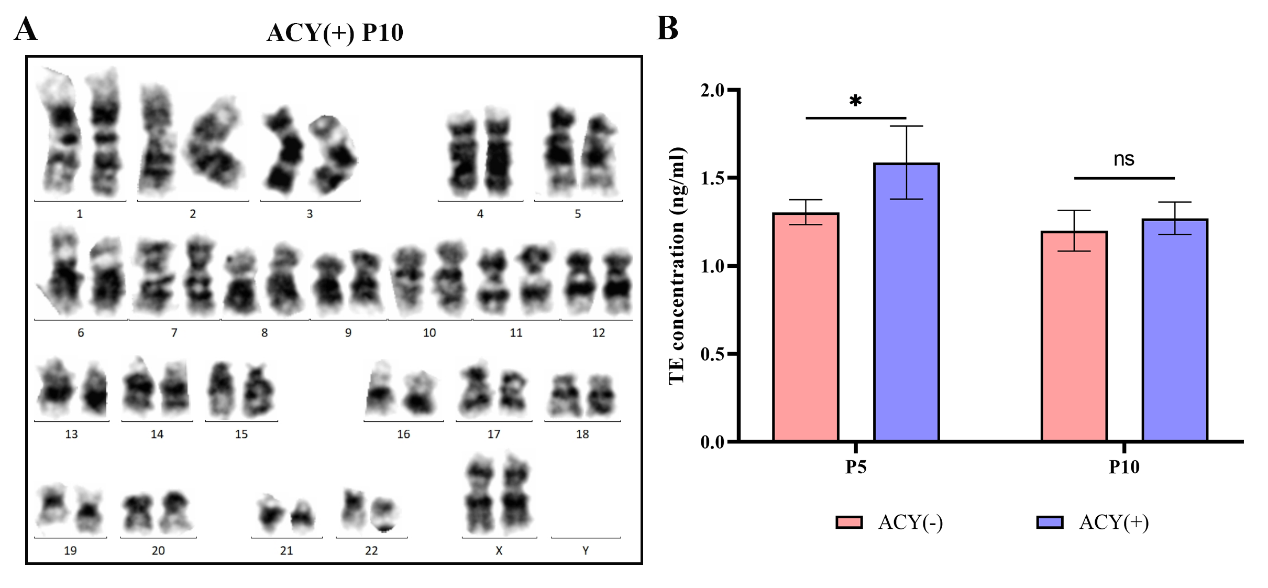


**Figure S8. Karyotype and telomerase activity analysis of MSCs.** (A) G-band karyotyping of the MSCs (passage 10th) maintained in ACY treatment. (B) Telomerase activity analysis at each passage, n = 5 per group. Data are shown as mean ± SD. *P < 0.05, and ns. means no significant.

**Table S1. Information of MSC donors**

| **Tissue type** | **Sex** | **Age** | **Race** | **Potential disease** |
| --- | --- | --- | --- | --- |
| Fat tissues | Female | 31y | Chinese | Healthy |
| Bone marrow aspirates | Male | 33y | Chinese | Healthy |
| Umbilical cord | Female | Newborn | Chinese | Healthy |

**Table S2. Primers used for the amplification of transcripts by qPCR**

| **Genes** | **Forward sequence** | **Reverse sequence** |
| --- | --- | --- |
| GAPDH | 5’- GTCTCCTCTGACTTCAACAGCG-3’ | 5’-ACCACCCTGTTGCTGTAGCCAA-3’ |
| RUNX-2 | 5’-CCCAGTATGAGAGTAGGTGTCC-3’ | 5’-GGGTAAGACTGGTCATAGGACC-3’ |
| Osteocalcin | 5’-CGCTACCTGTATCAATGGCTGG-3’ | 5’-CTCCTGAAAGCCGATGTGGTCA-3’ |
| ITBA2 | 5’-CAAAGGGTGGTTGGGAAGGATC-3’ | 5’-AGTTGATGACGGAAATTCGGAGC-3’ |
| PPARG | 5’-AGCCTGCGAAAGCCTTTTGGTG-3’ | 5’-GGCTTCACATTCAGCAAACCTGG-3’ |
| FABP4 | 5’-ACGAGAGGATGATAAACTGGTGG-3’ | 5’-GCGAACTTCAGTCCAGGTCAAC-3’ |
| ADIPOQ | 5’-CAGGCCGTGATGGCAGAGATG-3’ | 5’-GGTTTCACCGATGTCTCCCTTAG-3’ |
| Aggrecan | 5’-AGTCACACCTGAGCAGCATC-3’ | 5’-TCTGCGTTTGTAGGTGGTGG-3’ |
| COL2A1 | 5’-CCTGGCAAAGATGGTGAGACAG-3’ | 5’-CCTGGTTTTCCACCTTCACCTG-3’ |
| SOX9 | 5’-AGGAAGCTCGCGGACCAGTAC-3’ | 5’-GGTGGTCCTTCTTGTGCTGCAC-3’ |
| CCL2 | 5’-AGAATCACCAGCAGCAAGTGTCC-3’ | 5’-TCCTGAACCCACTTCTGCTTGG-3’ |
| TGF-β1 | 5’-TACCTGAACCCGTGTTGCTCTC-3’ | 5’-GTTGCTGAGGTATCGCCAGGAA-3’ |
| IDO | 5’-GCCTGATCTCATAGAGTCTGGC-3’ | 5’-TGCATCCCAGAACTAGACGTGC-3’ |
| HGF | 5’-GAGAGTTGGGTTCTTACTGCACG-3’ | 5’-CTCATCTCCTCTTCCGTGGACA-3’ |
| TSG-6 | 5’-TCACCTACGCAGAAGCTAAGGC-3’ | 5’-TCCAACTCTGCCCTTAGCCATC-3’ |
| CCL7 | 5'- ACAGAAGGACCACCAGTAGCCA-3' | 5'- GGTGCTTCATAAAGTCCTGGACC-3' |
| CCL8 | 5'- TATCCAGAGGCTGGAGAGCTAC-3' | 5'- TGGAATCCCTGACCCATCTCTC-3' |
